# Understanding the Evolution of Government Attention in Response to COVID-19 in China: A Topic Modeling Approach

**DOI:** 10.3390/healthcare9070898

**Published:** 2021-07-15

**Authors:** Quan Cheng, Jianhua Kang, Minwang Lin

**Affiliations:** School of Economics and Management, Fuzhou University, Fuzhou 350108, China; chengquan@fzu.edu.cn (Q.C.); n190720160@fzu.edu.cn (J.K.)

**Keywords:** COVID-19, pandemic, policy change, government attention, text mining, topic evolution path, China

## Abstract

The effective control over the outbreak of COVID-19 in China showcases a prompt government response, in which, however, the allocation of attention, as an essential parameter, remains obscure. This study is designed to clarify the evolution of the Chinese government’s attention in tackling the pandemic. To this end, 674 policy documents issued by the State Council of China are collected to establish a text corpus, which is then used to extract policy topics by applying the latent dirichlet allocation (LDA) model, a topic modelling approach. It is found that the response policies take different tracks in a four-stage controlling process, and five policy topics are identified as major government attention areas in all stages. Moreover, a topic evolution path is highlighted to show internal relationships between different policy topics. These findings shed light on the Chinese government’s dynamic response to the pandemic and indicate the strength of applying adaptive governance strategies in coping with public health emergencies.

## 1. Introduction

In recent decades, the world community has suffered many epidemics, such as SARS, MERS, Ebola, and Zika; therefore, effectively minimising the negative impact of these epidemics has become a global governance problem. Many have acknowledged that good governance or policy capacity is necessary during a crisis [1,2,3]. Large-scale public health emergencies triggered by pandemics require governments to have agile and adaptive governance capacities to control them quickly. Unfortunately, governments react similarly to ducks caught in a thunderstorm when faced with a pandemic, even though well-rehearsed public health emergency preparedness plans may be in place [4,5]. COVID-19 broke out in late December 2019, creating varied and distributed challenges to every country’s public health emergency system.

COVID-19 is characterised by swift transmission, glaring infection rate, and higher mortality rate than previous pandemics. There is little knowledge concerning how the Chinese government prioritised its efforts to combat the disease, although Chinese people have experienced similar pandemics in the past. It has been posited that one feature was that COVID-19 policies changed promptly and quickly [6]. In practice, however, few countries or regions could contain COVID-19 within a short time. One global public health expert said, “knowing how to control the spread of coronavirus is not rocket science, but actually doing it has proved elusive and difficult for many governments across the world” [7].

China is the best research sample for comparing policy responses at the country level. As the first country severely hit by the COVID-19 disease, China successfully tamed the uncontrolled spread of the disease within two to three months and gained plenty of experience in fighting against COVID-19 [8,9]. China’s prompt responses and coordinated actions are described as its governance model [10].

To date, a growing number of studies have attributed China’s success in containing COVID-19 to specific actions carried out by the central government, such as real-time information delivery [11], aggressive quarantine measures [12], national human mobility restrictions [13], strict community lockdown [14], knowledge-driven scientific decision making [15], and official sanctions [14]. With a different tradition, the Chinese central government has strong coordination and mobilisation capacities due to its multilevel authoritarian governance structure and centralised leadership. Some scholars argue that national coordination, especially intergovernmental coordination, plays a crucial role in containing the spread of COVID-19 [14,16,17]. Strong national coordination is reflected in resource scheduling and allocation and information sharing over a short period of time [11,18]; however, during pandemic emergencies, government attention is a scarce resource, meaning policymakers cannot pay equal attention to all specific responses. As such, questions remain regarding whether the Chinese government have adjusted its policies and attention during the COVID-19 control process, and how the government prioritised its responses in such a short time. Although some scholars have focused on policy adjustment regarding China’s efforts to control COVID-19 [14,16], there is still no clarity about the underlying dynamic mechanism. In addition, most related studies were not written as research-based articles but as commentaries or reviews based on the authors’ intuitive knowledge.

This article aims to fill the research gap to provide more robust empirical evidence with a more rigorous and scientific approach. It seeks to reveal how the Chinese government changed its policies (attention and action) in response to the COVID-19 pandemic, which other countries can draw experience. In detail, COVID-19 control policies issued by the State Council of China from 20 January 2020 to 9 September 2020 were collected to establish a text corpus with 674 documents. Then, the policy corpus was fed to the latent dirichlet allocation (LDA) model, a topic training model, in order to dynamically analyse the different government attention focuses during each period of COVID-19 control, map the evolution of the government’s attention allocation through a vivid and concrete picture, and demonstrate the policy change mechanisms underlying the response to the spread of COVID-19.

The rest of this article is organised as follows. Section 2 provides a review of related literature on COVID-19 control and government responses to public health crises. The data description, methodology, and empirical analysis results are presented in Section 3 and Section 4. A discussion of the results follows in Section 5, which also presents findings derived from the Chinese experience that the international community and policymakers can learn from. The conclusions are presented in the final section.

## 2. Literature Review

Previous studies on COVID-19 have focused on containing the spread of the disease and relieving the negative impacts, including the necessity and effectiveness of lockdown policies [19], which China was the first country to implement. Many scholars have pointed to the economic recessions triggered by the lockdown and quarantine policies [20]; however, given the evidence in China, containment strategies should include lockdown and quarantine policies in order to reduce transmission rates [21]. Another important policy to emphasise is the promotion of economic growth. An empirical study found that macroeconomic downturns have a negative impact on infectious disease mortality rates [22]. The efficiency of a country’s health expenditure can help to explain why China could quickly and successfully contain COVID-19. Since the middle of the 1990s, China has increased healthcare spending, which is a critical predictor of healthcare system performance [23]. A strong healthcare system is important in controlling COVID-19. At the beginning of the COVID-19 outbreak, medical care resources played a significant role in containing the disease [24]. In general, China’s experience in fighting COVID-19 suggests that strong government intervention is an efficient strategy during a pandemic.

Actually, “COVID-19 forces us to rethink governance” [16]. The study on emergency-based governance is generally conducted in three approaches. The first group of scholars focuses on the agent level (e.g., street-level bureaucrats [25], community-based organisations [8], non-profit organisations [26,27], citizens [28,29,30]). They emphasised the value of involving multiple stakeholders (public, private, and non-profit sectors) and the importance of democracy. The Chinese experience also provides strong evidence that taking collaborative actions can help curb the spread of COVID-19 [31]. The second group of scholars largely works on the political and institutional levels. Good or agile leadership is regarded as a valid approach to dealing with pandemics [32,33]. Though political leadership plays an essential role in fighting pandemics [34], countries’ political and institutional differences shape their emergency responses [33,34,35,36,37,38]. Partisan politics also matters [39]. A study found that democratic governors and governors without term limits responded faster to containing the pandemic in the US [40]. Political support enhances the government’s capacity for handling the pandemic [3]. The third group of scholars examines the government response to COVID-19 using a normative approach. For example, Cairney assessed the UK government’s COVID-19 policy and recommended that the government use a trial and error strategy during a pandemic [6]. Mazey and Richardson (2020) argue that anticipatory policy making is more effective than reactive policy making. While many scholars call for rethinking government function and governance resilience [41,42], so far, the terrain of these previous studies on government response to COVID-19 is still largely fragmented. The existing literature pays little attention to the dynamic of crisis governance in the face of COVID-19.

Most scholars prefer to find out the defects in policy responses by central and local governments and propose suggestions regarding how to curb the pandemic. Only a few studies reflected on pandemic governance from a dynamic perspective. More specifically, limited scholarship has focused on policy change or attention shift during the different periods of COVID-19 control. Simon (1947) introduced attention to administrative behaviour research, which provides a new theoretical perspective for illustrating decision making in administrative organisations. He defines attention as a process in which managers selectively focus on some information and ignore others. Attention is deemed a scarce resource [43], as policymakers cannot pay equal attention to all public affairs due to time and cognitive limitations. In particular, what policy response can get policymakers’ attention, to some extent, depends on how policymakers prioritise the policy responses within their cognitive limitation. This priority-making process is also described as attention allocation. Attention allocation in governments has evolved as an analytical approach to explain policy responses to external shocks. Because the limitation of human cognition prevents adequate policy attention from being drawn to respond to environmental changes, there is not enough time for policymakers to cycle through all the possible solutions during a crisis. Therefore, only a few options will get the attention of policymakers. Moreover, governments would not focus on only one policy option during a crisis due to the rapidly changing environment. In other words, in the face of rapid environmental change, there is indeed no one-size-fits-all policy for successful emergency governance [44]. Selective attention influences political choice. The pandemic has demonstrated that various response strategies are needed to ensure policy responses fit with the environment [45].

How can one better understand policy change in response to public health crises? Brian Jones (1994) proposed an attention-driven policy choice model that pointed out that a policy change is contingent on government attention allocation [46]. Given the fast and global spread of COVID-19, governments must adjust attention over time in line with changing situations. The shift in attention will be reflected in the direct change in policy response. However, there is still no clarity on how policy changes to keep pace with the changes during a crisis. Adaptive governance, originating from the evolutionary theory, stresses learning as the core of governance efforts [45]. It implies that there exist implicit learning dynamics among different policy responses. In the long term, the evolutionary dynamics of policy changes would become discernible [47].

The pandemic, however, must be brought under control in a short notice. This makes it difficult to identify the dynamics of policy change. Fortunately, using the lens of government attention allocation can help us easily grasp nuanced policy response changes during a pandemic. Policy responses will be adjusted along with the shift in the focus of government attention. By studying the evolution of government attention, we can deduce the presence of learning links among policy response strategies.

## 3. Materials and Methods

### 3.1. Research Design

**Figure 1:**
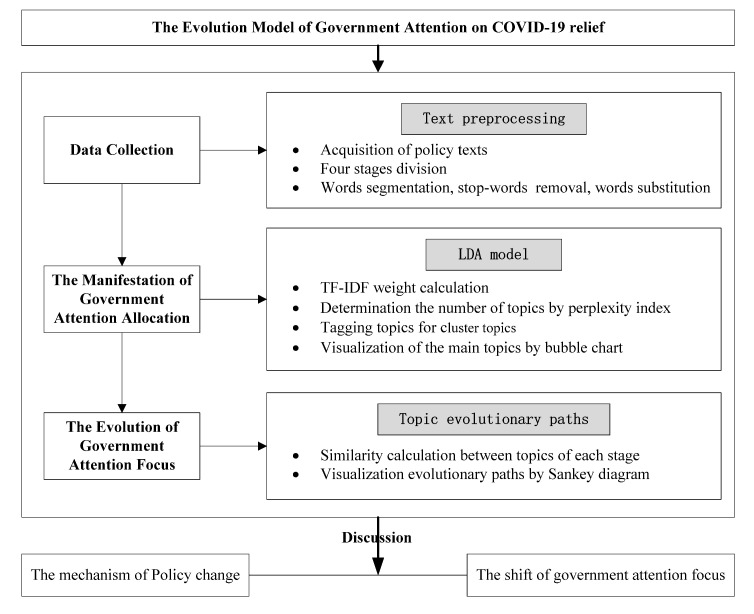
Evolution model of government attention on COVID-19 relief.

According to the theory of government attention allocation, the government pays different levels of attention to the pandemic prevention strategies as events unfolded. Therefore, it would be a great perspective to study government attention allocation and policy change in crisis governance combined with government attention. Based on a topic evolution model, this article builds the policy evolution model of Chinese central government attention on COVID-19 relief. The research subject is divided into three parts: data collection, the manifestation of government attention allocation, and the evolution of government attention focus. Furthermore, the specific research process is shown in Figure 1.

### 3.2. Four Stages of COVID-19 Pandemic Management in China

**Figure 2:**
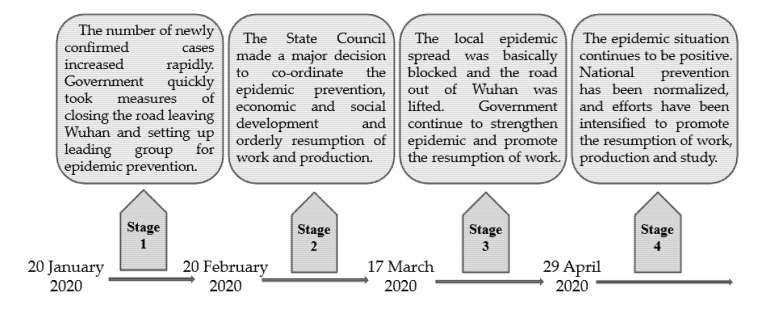
Four stages of managing the COVID-19 pandemic in China.

On 7 June 2020, the State Council Information Office of the People’s Republic of China released a White Paper, “The China Action to Combat the COVID-19 Pandemic”, in which it divides the timeline of the Chinese government’s efforts to combat the COVID-19 pandemic into five periods according to the spread status of the virus. However, the Chinese central government issued the first policy document regarding COVID-19 management in the second period. To explore the policy change, we only focused on the last four periods and renamed them “Stage 1”, “Stage 2”, “Stage 3” and “Stage 4,” respectively (see Figure 2). Stage 1, from 20 January 2020 to 20 February 2020, features preliminary containment of COVID-19. The milestone event during this stage was the lockdown of Wuhan city. Stage 2, from 21 February 2020 to 17 March 2020, features initial control of COVID-19. A symbolic event of this stage is the drop in the number of new local COVID-19 infections to single digits. Stage 3, from 18 March 2020 to 28 April 2020, features overall control of COVID-19. On 18 March 2020, China reported zero new local cases for the first time. Stage 4, from 29 April 2020 to 9 September 2020, features regular COVID-19 prevention and control process. Since 29 April 2020, the Chinese central government has made new arrangements to implement regular COVID-19 prevention and control measures and fully advance work resumption.

### 3.3. Data Collection

The data used in this article are policy documents issued by the State Council of China which are available in the public domain exempt from ethical approvals. Since the outbreak of COVID-19 in late December 2019, the Chinese central government has issued many policy documents in response to the negative impact of the virus’s rapid spread. Policy documents serve as the “carrier” of commands that symbolise policymakers’ intentions or ambitions. Thus, policy documents provide us a good channel to analyse policy change [48]. All the policy document data were extracted from the Chinese central government official website (http://www.gov.cn/, accessed on 10 September 2020) hosted by the General Office of State Council of China. Policy Document Database is one of the modules of this website. “Pandemic prevention and control”, “joint prevention and control” and “work resumption” were the search terms used to anchor the target policy documents published between 20 January 2020 and 9 September 2020, which have been categorised into four stages. A total of 674 policy documents at the central government level were extracted after screening out duplicates and irrelevant data. More specifically, the number of policy documents in the first stage was 178, while the second, third, and fourth stages had 142, 145, and 209 documents, respectively.

### 3.4. Methods

The Latent Dirichlet Allocation (LDA) topic model, a text mining technique, is employed for this study. To begin with, we conduct a two-procedural text reprocessing. The first process involves a word segmentation of the 674 policy documents and creating a self-defined word segmentation dictionary. After that, a screening thesaurus established by Harbin Institute of Technology-Baidu is employed to eliminate unneeded words, and a policy text corpus is extracted from the policy documents. The second process involves vectorizing the policy text corpus with the bag-of-words technique, and then applies the TF−IDF (Term Frequency-Inverse Document Frequency) statistical method to determine the weight of each term. TF−IDF weight is calculated as:

(1) TF−IDF(t,d)=TF(t,d)∗IDF(t)

where TF(t,d) represents the frequency of term t appearing in the document d. IDF(t) refers to inverse document frequency, which is used to determine the importance of the term t in expressing semantic dimension. It is calculated as follows:

(2) $$IDF(t)=\log\frac{Total\;number\;of\;documents}{Number\;of\;documents\;containing\;t+1}$$

In other words, only the distinguishing terms are picked to represent the true meaning of documents, which means a selected term would appear more frequently in certain documents but less frequently in others.

**Figure 3:**
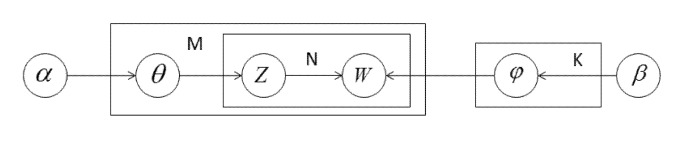
LDA topic model.

Subsequently, we use the LDA topic model to reveal the potential relationship between the topics extracted from the different policy documents. David Blei (2003) proposes the LDA topic model, a document-topic model based on a three-level hierarchical Bayesian model, which applies an unsupervised text-mining technique to pick up potential topics from initial documents [49]. The LDA model consists of a three-level structure of words, topics, and documents and can extract potential topics and the probability distribution of each topic from a large number of texts [50]. Figure 3 shows the specific operating process of the LDA topic model. First, it generates “document-topic” probability distribution *θ* from document sets *M* and “topic-keyword” probability distribution *φ* from topic sets *K*, and then extracts specific topics *Z* and corresponding words *W* from a vocabulary set *N*, wherein both *θ* and *φ* are Dirichlet distributions containing the priori superparameters, *α* and *β*.

It is vital to determine the optimal number of topics to map out the distribution of topic intensity and topic evolution path. To this end, we employed a perplexity index based on the LDA to train the model to confirm the number of policy topics automatically. The perplexity index is calculated as follows:

(3) $$Perplexity=exp\left(-\frac{\sum_{d=1}^{M}log_{2}p\left(w_{d}\right)}{\sum_{d=1}^{M}N_{d}}\right)$$

where M is the number of documents, $N_{d}$ is the term dataset of each document, and $P(w_{d})$ represents the probability of each term generated in a training model.

Based on the aforementioned mathematical methods, we employ Python 3.0 to extract the core topics in the policy documents coming from each stage of managing the COVID-19 pandemic in China, and visualised the results using a bubble chart to display the distribution and intensity of core topics in each stage. Then, we continue to trace the evolution of core topics in each stage and map them using a Sankey diagram.

## 4. Results

### 4.1. Manifestation of Government Attention Allocation on COVID-19 Relief

In order to detect the core topics in the policy documents that explain how the government shift its attention during COVID-19, we employ Python 3.0 to do the LDA topic modelling. Learning from the method proposed by Griffiths and Steyvers [51], we adopt Gibbs sampling technique, then set the hyper-parameter (α and β) of the LDA training model as 50/K and 0.1 and set the iteration times as 1000. Two important input parameters of the LDA model are the document-topic matrix and the number of topics k. A document-topic matrix can be obtained by calculating the TF-IDF weight of the policy text thesaurus acquired during the text preprocessing. The number of topics $k_{i}\left(i=1,2,3,4\right)$ can be determined by calculating the perplexity index. After repeated attempts, the number of core topics is determined to be 7 in the first stage, 6 in the second stage, 9 in the third stage, and 7 in the fourth stage. After that, we continue to analyse topics of the policy text thesaurus by using the LDA model. The procedure is as follows: firstly, calculating the main word clusters appearing in policy documents; secondly, clustering similar words to form some specific topics, then calculating the weight of each word in corresponding topics, and confirming each document belongs to a specific topic. Based on the aforementioned methods, we strive to determine the main topics, numbers of documents, and tagged topics in each stage.

#### 4.1.1. Stage 1: Preliminary Containment of COVID-19

**Figure 4:**
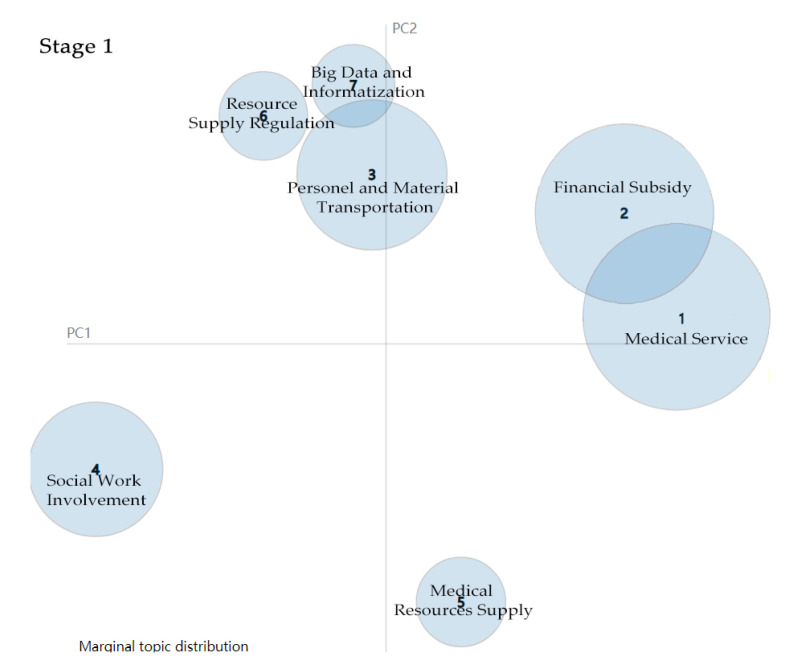
Topic intensity in Stage 1.

In the first stage, the soaring COVID-19 infection rate forced central government to pay much more attention to COVID-19 containment. Table 1 shows the LDA topic cluster result based on 178 policy documents issued by the Chinese central government in the first stage. We find that it devotes itself to providing medical-related services and ensuring the supply of medical materials is the Chinese central government’s priority. We then calculate the topic-word distribution probability using the LDA topic training model and further visualise the result using a bubble chart. The size of the bubbles indicates the intensity variation of main topics (see Figure 4). We find that medical service and financial subsidy catch most of the government’s attention. Concerning financial subsidy, the Chinese central government quickly took command to “increase capital investment and ensure the funding needs for all regions” after the outbreak of COVID-19. In mid-February, financial departments at all levels had allocated more than RMB90 billion to provide a strong guarantee for implementing the pandemic prevention and control plan. The other main topics concern how to provide better medical environments and support medical treatment. People’s lives were prioritised during the initial period. As a result, by 18 February 2020, the number of confirmed infections began to decline.

#### 4.1.2. Stage 2: Initial Control of COVID-19

**Figure 5:**
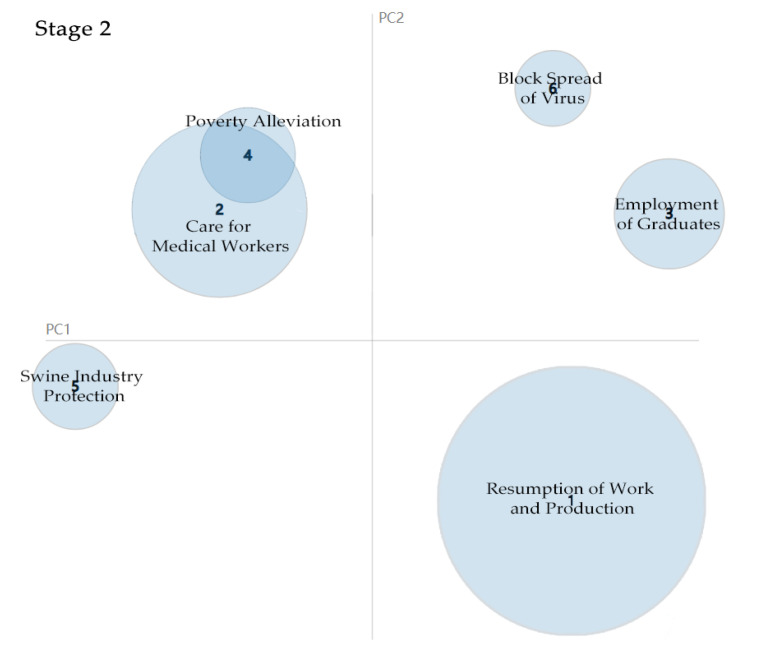
Topic intensity in Stage 2.

Six main topics are extracted from 142 policy documents in the second stage (see Table 2), mostly concerning how to mitigate the negative impact of COVID-19, such as providing sufficient jobs for graduates and rural workers. Since the number of new infections fell to single digits, the Chinese central government increasingly shifted its attention to work resumption. As shown in Figure 5, work resumption is the biggest bubble, which means the government paid the most attention to restoring its economy in the second stage. Based on the improving state of the pandemic, the Chinese central government launched a series of guidance policies to return life to normal. For instance, the Ministry of Industry and Information Technology released a policy document titled “Promote Work Resumption for Industrial and Communication Enterprises,” the Ministry of Transport issued one titled “Accurately and Orderly Resume Transportation and Steadily Promote Work Resumption,” while the Ministry of Science and Technology issued “Prevent COVID-19 scientifically in National High-tech Zones and Return Enterprises to Work in an Orderly Way”.

#### 4.1.3. Stage 3: Overall Control of COVID-19

**Figure 6:**
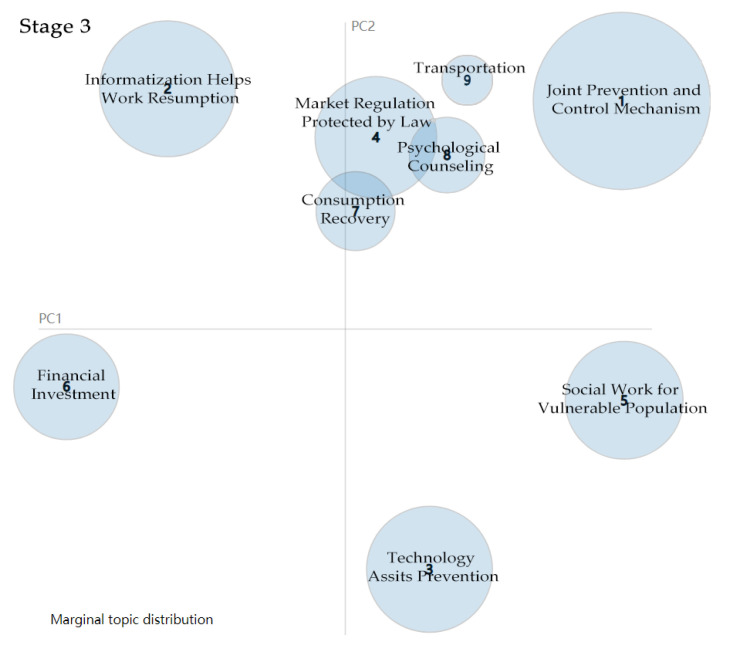
Topic intensity in Stage 3.

Nine topics are extracted from 145 policy documents in the third stage (see Table 3). Joint prevention and control mechanisms are the main topics, meaning the government does not focus on only medical treatment. Taking precautions against COVID-19 is a top-priority task since the number of new local cases dropped to zero in this stage. Therefore, prevention is necessary as the spread of COVID-19 has been completely contained. Moreover, the government alone cannot successfully contain COVID-19. The effort requires collaboration with multiple actors, such as NGOs, social workers, the citizens, and enterprises. From the result of the LDA training model, the high-frequency tagged words for joint prevention and control mechanism are transportation, nucleic acid testing, and social organisations, which show that the government tried to coordinate many resources in its bid to control the spread of COVID-19. By comparing Figure 5 and Figure 6, it can be seen that joint prevention and control has replaced work resumption to become the biggest bubble, which means the government has shifted its attention in the third stage.

#### 4.1.4. Stage 4: Regular Prevention and Control of COVID-19

**Figure 7:**
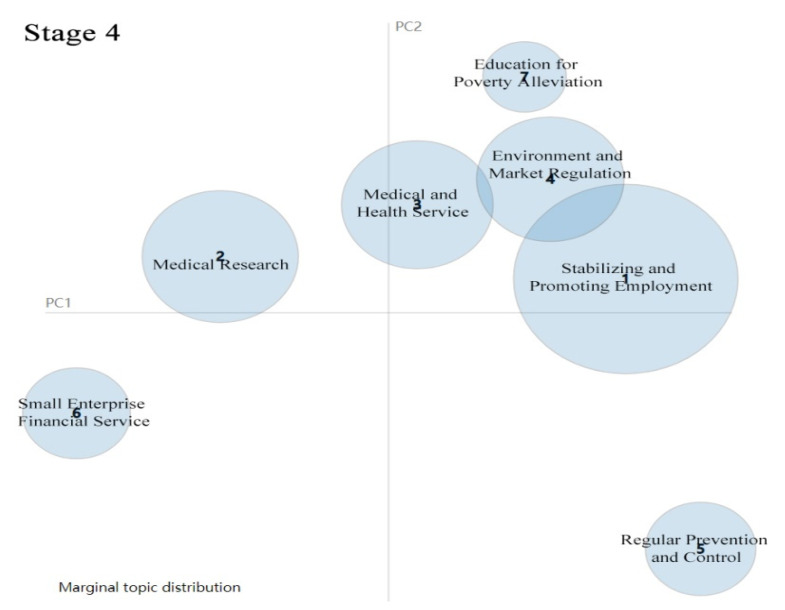
Topic Intensity in Stage 4.

After 29 April 2020, China had recovered from the COVID-19 outbreak, with life slowly returning to normal. The Chinese central government paid particular attention to ensuring employment for college graduates and laid-off and peasant workers. Thus, stabilising and promoting employment became the central topic in most policy documents (see Table 4). In this period, many regions in China adjusted their public health emergency response level to COVID-19. Figure 7 shows that stabilising and promoting are the biggest bubble, which means the Chinese central government has shifted its focus during the fourth stage. College graduation season is usually from May to September each year. The government took measures to ensure and promote employment for college graduates to avoid mass unemployment and enrich human resources in the labour market. All the efforts were attributed to economic recovery and the protection of people’s livelihood. 

### 4.2. Evolution of Government Attention on COVID-19 Relief

Based on the afore-discussed topic intensity analysis results, it can be deduced that the central government allocates its attention to different affairs at each stage of the COVID-19 eradication plan. In general, however, topic evolution is not static but a dynamic process. We continue to trace the topic evolution path used to reveal the relationship between topics and time dimension to reveal the dynamic mechanisms. Based on the topic-word probability distribution of the LDA topic model, we adopt topic similarity to identify the topic relevance among adjacent periods to map the evolution path of the main topics that appeared in the policy documents. In information theory, Kullback–Leibler Divergence (KLD) is generally used to describe the difference in two variables’ probability distributions. Because KLD lacks a symmetry attribute, we use the Jensen–Shannon Divergence (JSD) instead, whose value range is [0, 1]. The smaller the JSD value, the greater the eigenvector relevance between topics becomes. The JSD value is calculated as:

(4) $$JS(P_{1}||P_{2})=\frac{1}{2}KL(P_{1}||\frac{P_{1}+P_{2}}{2})+\frac{1}{2}KL(P_{2}||\frac{P_{1}+P_{2}}{2})$$

To better identify the evolving relationship between topics in each stage, we use the cotangent function to transform the JSD value to obtain a new value named $JS_{change}$, so that the topic similarity would positively correlate with $JS_{change}$ value.

(5) $$JS_{change}=\cot(\frac{\pi }{2}*JS(P_{1}||P_{2}))$$

After that, we use the Sankey diagram to visualise the similarity between topics in adjacent stages. We employ the Python 3.0 piechart library to draw a Sankey diagram based on the premise that the threshold of the average similarity between topics is 1.38. The results are shown in Figure 6, where the four columns represent the four stages of managing COVID-19, the element blocks in each column denote the main topics in each stage, their size symbolises the degree of similarity between topics in different stages, and the width of the lines between each column define the similarity value between topics.

**Figure 8:**
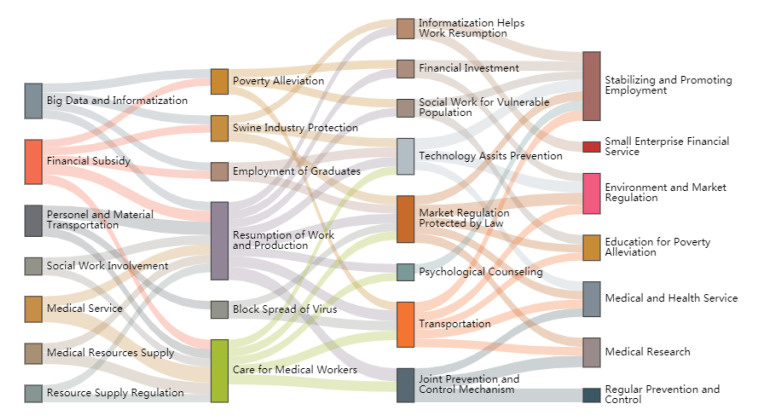
The topic evolution path of managing COVID-19 in China.

Figure 8 shows that the government’s attention evolves during the different stages, indicating that it changes its policy responses to fighting COVID-19. In the first stage, the Chinese central government allocated its attention to specific policy responses, such as medical services, financial support, personnel and material supply and transportation, social work, and information disclosure, devoted to containing COVID-19 and returning life to normal. All these policy responses evolved into other policy responses in the next stage. Policy efforts in the first stage are all related to work resumption in the second stage. As mentioned above, the spread of COVID-19 was cut off in the second stage, which led to the Chinese central government considering whether or not to adjust its policy response. The application of big data, subsidy of financial funds, transportation, and material supply in the first stage were all to prepare for the resumption of work. For example, financial subsidies evolved into poverty alleviation, college graduate employment, work resumption, and care for medical workers. Work resumption evolved into government efforts in the third stage, and joint prevention and control mechanisms were highly correlated with the two main policy responses in the second stage: work resumption and care for medical workers. During the fourth stage, COVID-19 in China was under control. Employment support drew most of the government’s attention. The Chinese central government views employment as the foundation of people’s wellbeing and development. It can be deduced from Figure 6 that promoting stable employment is related to most policy efforts in the previous stage.

## 5. Discussion

This article harnesses a topic modelling approach to identify the topic intensity and evolution path of COVID-19 relief efforts from data collated from the Chinese central government’s policy documents. This is to investigate how and in what ways the Chinese government responds to the COVID-19 crisis. When a natural disaster or accident triggers a crisis, the government’s primary concern is the availability of an emergency preparedness plan. If the answer is yes, then it is implemented. However, the uncertain and uncontrollable development of COVID-19 paralyses governments’ pre-existing emergency plans. Thus, it calls for a more robust governance strategy that facilitates adaptive and flexible adjustments [52]. The policies need to change in response to the situational changes in the crisis [53]. By using adaptive governance theory, we illustrate how the government has shifted policy attention during the pandemic.

### 5.1. Adaptive Governance Explains Why Government Shifts Attention Focus

As mentioned before, the inability of pre-existing emergency plans to deal with the outbreak of COVID-19 is normal, as it was a novel disease and the crisis demanded real-time and evolving policy responses. Concerning crisis governance, public administration scholars suggest that governments need to establish prompt decision-making systems for quick responses [54]. They should also be more flexible and agile to adapt to new and emerging problems [52]. This suggestion aligns with the tenet of adaptive governance, which is mainly characterised by regarding adaptation as a response to the environment over time [45]. Adaptive governance drives governments to make suitable policies that fit changing environments quickly until the crisis is resolved. For an unpredictable crisis such as a pandemic, an adaptive governance strategy is needed [55].

As the pandemic unfolds, we observe the different policy tools used by the government to mitigate its negative impacts. We also notice that policy responses and allocation of government attention to the pandemic are not static. Therefore, we argue that the adaptive approach is the most useful crisis governance strategy to fight a pandemic. Adaptive governance in emergency response recognises cognitive limitations since the full array of tasks cannot be identified in advance [56]. It plays a critical role in dealing with an unpredictable crisis [55]. However, it is difficult to understand what adaptive crisis governance is for lack of specific context.

This study reveals that the Chinese central government has shifted its attention during the different stages of managing COVID-19 by analysing the topic intensity of policy documents. The pandemic is not static, as it spreads fast and creates a rapidly evolving health crisis in China. The government’s attention is a scarce resource due to the cognitive limitation of policymakers, which results in the central government selectively focusing on significant responses. The government’s shift of attention during the different stages can be classified as an adaptive governance strategy. It is agile and adaptive to contain COVID-19 and reduce its negative impacts on people’s daily lives. Many scholars point out that the European Union’s and Indonesia’s emergency management of COVID-19 involved a certain degree of adaptability [57,58].

Scholars have mostly agreed that pandemic emergency management calls for robust governance solutions that are adaptable [52], and adaptive governance allows for the adjustments needed to cope with shifting conditions [56]. Therefore, governments should be prepared to react to the dynamic nature of the pandemic. As the government gradually contains the pandemic, it is responsible for restoring normalcy. The changing efforts taken by the government are in line with the development of the pandemic. Adaptive governance requires the government to build a decision mechanism adaptive and resilient to new or unexpected situations [59]. China built its adaptive response system after the SARS outbreak in 2003 and implemented it in the aftermath of the Lushan Earthquake in 2013 [60]. The resilience of the Chinese institution, particularly in crisis governance situations, has received many compliments. Hence, it is no surprise that the Chinese central government shifted its focus during the COVID-19 control period in a steady and robust manner.

### 5.2. Policy Change Mechanisms during the Short Crisis Control Periods

Although research focusing on policy change in crisis governance exist, most of their findings are based on a long history, not on short periods. Over a long time, policy change dynamics can become discernible [47,61]. For example, Moloney analysed Australian quarantine policy changes across 120 years and concluded that policy shifts were crucial to successful COVID-19 response [62]. Alternatively, we can identify policy change in two discontinuous crisis events [61]. However, the COVID-19 pandemic requires quick government responses during each stage of its spread. Therefore, it is hard to observe the nuanced policy changes and identify their mechanisms during such a limited period. To date, whether, and to what extent, the government will change policies in response to the pandemic remains an open question. Kettle posits that solving COVID-19 will require policy changes [53]. This study confirms that government would change its policy decision to adapt to a new situation caused by the spread of the disease and resume people’s daily lives as soon as possible.

First, policy changes can be predicted by the shift of the government’s attention. Government attention allocation helps us comprehend the intent and action of policy changes during the pandemic crisis, which also means we can judge whether or not the government change policy by identifying the shift of government’s attention and reveal the trend of policy change through mapping the evolution of government’s attention across different stages. The finding resonates with the attention-driven policy choice model proposed by Brian Jones [46]. Second, policy changes can be caused by external shock. External perturbation is a necessary condition for major policy change [63]. During a pandemic, the spread of the disease is an external event that significantly impacts the government’s decisions. We find that the policy changes were in response to the spread of COVID-19, which provided additional evidence for further understanding the link between external events and policy changes. Third, the policies across the different pandemic management stages have intrinsic relationships. Nohrstedt and Weible introduce the concept of policy proximity to explain the underlying logic of policy changes during a crisis [64]. During the COVID-19 crisis, the policies in response across the different stages are interconnected. Learning plays a big role in crisis decision making because of the limited time available to policymakers, as underscored in many policy change scholarships [44,65]. One policy in the previous stage would evolve into another policy in the next stage. This means governments should draw up strategic emergency plans during the early stages of a crisis.

### 5.3. Practical Implications

Through the text mining of the topic intensity and topic evolution path based on policy documents issued by the Chinese central government during the COVID-19 control period, we effectively verify that the central government’s attention during emergencies is allocated to different but related topics to adapt to shifting conditions during the pandemic. This shift in attention is accompanied by changing policy efforts. The Chinese central government’s policy efforts are classified into five distinctive aspects: information-based decision, healthcare priority, transportation assistance, financial support, and social work supplement, which point out the right direction for pandemic prevention and control.

Firstly, supporting policy decisions with big data. In the evolution of policy topics, the application of big data ran through all stages of managing COVID-19. Big data application supports various policy decisions. For example, intelligent transport big data from the Baidu Map aided work and production resumption decisions.

Secondly, building an adaptive public health emergency system. The Chinese government’s attention on healthcare was adjusted as the pandemic evolved. During the initial stages, the Chinese government gathered medical resources to treat patients and control the spread of the disease.

Thirdly, setting up flexible transportation systems. During the pandemic, the government needed to ensure the traffic work normally. At the initial stages, the Chinese government set up “green channels” to ensure enough medical resources and daily necessities. In the later stages, the government paid attention to traffic control to ensure people can return to work and school.

Fourthly, establishing an agile financial subsidy policy. In light of the risks associated with pandemics, a financial support policy is necessary. The Chinese government provided many innovative financial policy tools, such as low-interest loans and consumption coupons, to support enterprises and stimulate consumption.

Lastly, strengthening community services with social workers. Mass prevention and control were some of the most important policy tools adopted by the Chinese central government in response to COVID-19 containment. It required citizen participation in community public health emergency work. The Chinese experience heavily relied on social workers, who played a significant role in combating the spread of COVID-19, particularly by providing community services to the vulnerable population.

### 5.4. Limitation

The present study has several limitations. First, this study analyses government attention and policy changes based on policy documents. However, the selected policy documents do not cover all the government decision-making efforts. Many government decisions not in the selected policy documents are neglected, which has implications for our conclusion. However, most decisions on fighting COVID-19 made by the Chinese central government are directly presented in policy documents and published openly for the public because information disclosure is a basic requirement, particularly during the pandemic emergency period.

Second, while the text content analysis methodology is widely used in social science research, it is criticised for its high subjectivity. That means the analysis results are easily disturbed and distorted by the subjective intent of the researcher. However, to avoid this problem as much as possible, two of the authors operate the process and coding separately, then discuss and resolve the inconsistent results with the third author.

Third, although this study employs adaptive governance theory to explain the attention focus shift and speculated the mechanism of policy change by observing the topic evolution path, it remains unclear whether, and to what extent, policy changes in this manner from the perspective of politicians and policymakers. Thus, additional evidence is needed to support our research findings.

We suggest collecting from other data sources to build a more rigorous and robust research inquiry in future research. These include interviewing policymakers to obtain qualitative evidence to support the findings, analysing the emotional feedback of the media and the public on the emergency actions taken by the central government. Moreover, the research question can be studied at the local government level to explore the differences between central and local governments.

## 6. Conclusions

In conclusion, this study employs text mining and content analysis methods to explore how the government changes policy in response to the pandemic. It explores the focus of government attention and the dynamic evolution path in each COVID-19 prevention and control stage based on the policy documents issued by the Chinese central government. First, we pick up the main topics of policy documents and extract the cluster topics of four COVID-19 prevention stages. We conclude that the government has paid attention to different response policies at different stages. Then, we analyse the evolution of the Chinese central government’s attention using topic intensity distribution and topic evolution path. The results show that the main topics in each stage are linked to each other. Finally, we discuss the research findings from two theoretical perspectives: adaptive governance theory and policy change theory. In addition, we conclude that there are five practical implications from the Chinese central government efforts that can help with public health emergency management in other countries. Knowing how the government responds to pandemic emergencies is of practical significance in improving the national public health emergency management system. These findings are expected to help us reflect on the use of adaptive governance strategies during a crisis.

## Figures and Tables

**Table 1:** Topic contents in Stage 1.

Cluster Topics	Number of Documents	High-Frequency Tagged Word
1–1 # Medical Service	47	[‘Medical Staff’, ‘Hospital’, ‘Health Department’, ‘Maternal’, ‘Informatization’, ‘The Ministry of Finance’, ‘Administration’, ‘Public Health’, ‘Fever Clinic’, ‘Medical Treatment’]
1–2 # Financial Subsidy	43	[‘The Ministry of Finance’, ‘The Central Government’, ‘Fund Guarantee’, ‘Necessaries of Life’, ‘Financial Institutions’, ‘Financial Fund’, ‘Uses of Funds’]
1–3 # Personal and Material Transportation	35	[‘Transportation’, ‘Human Resource‘, ’Peasant Worker’, ‘Health’, ‘Social Security’, ‘Disinfectant’, ‘Work Resumption’, ‘Staff’, ‘Wear Mask’]
1–4 # Social Work Involvement	17	[‘The Elderly’, ‘Nursing Facility’, ‘Human Resources’, ‘Civil Affairs’, ‘Department of Social Security’, ‘Mental Crisis Intervention’, ‘Vocational Training’]
1–5 # Medical Resources Supply	16	[‘Protective Clothing’, ‘Protective Equipment’, ‘Goggles’, ‘Tax Institution’, ‘Taxpayer’, ‘General Administration for Customs’, ‘Value-added Tax’, ’License’]
1–6 # Resource Supply Regulation	13	[‘Unlawful Act’, ‘Regulators’, ‘Control material’, ‘Operator’, ‘Medical Equipment’, ‘Market Supervision’, ‘Inspection’, ‘Crackdown’]
1–7 # Big Data and Informatization	7	[‘Information Technology’, ‘Informatization’, ‘Big Data’, ‘Network Security’, ‘Supply Chain’, ‘Digitalization’, ‘Internet’, ‘Industry Chains’]

**Table 2:** Topic contents in Stage 2.

Cluster Topics	Number of Documents	High-Frequency Tagged Word
2–1 # Resumption of Work and Production	80	[‘Work Resumption’, ‘Civil Affairs’, ‘Broadcast Television’, ‘Joint Prevention and Control’, ‘Health Department’, ‘Peasant Worker’, ’Economic and Social’, ‘Human Resource’]
2–2 # Care for Medical Workers	30	[‘Medical Staff’, ‘Ministry of Finance’, ’Health Department’, ‘Hospital’, ‘Intellectual Property’, ‘Social Security’, ‘Human Resource’, ‘Psychological Assistance’]
2–3 # Employment of Graduates	14	[‘College Graduates’, ‘Public Institution’, ‘Human Resources’, ‘Ministry of Finance’, ‘Institution of Higher Education’, ‘Ministry of Education’, ‘Agricultural Rural’, ‘Ministry of Agriculture and Rural Affairs’]
2–4 # Poverty Alleviation	8	[‘Poverty Alleviation’, ‘Relocation of Impoverished Residents’, ‘Labor’, ‘National Development and Reform Commission’, ‘Public Service’, ‘Work Resumption’, ‘Leading Group’, ‘Pairing Assistance’]
2–5 # Swine Industry Protection	6	[‘Private Enterprise’, ‘Swine Production’, ‘Farm’, ‘African Swine Flu’, ‘Work Resumption’, ’Agriculture & Rural Area’, ‘Harmless’, ‘National Development and Reform Commission’, ‘Industrial development’]
2–6 # Block Spread of Virus	4	[‘Wildlife’, ‘Transportation’, ’Road Transport’, ‘Management’, ‘Masses’, ‘Competent Department’, ‘Animal Epidemic Disease’, ‘Animal Epidemic Prevention’, ‘Railways Bureau’]

**Table 3:** Topic contents in Stage 3.

Cluster Topics	Number of Documents	High-Frequency Tagged Words
3–1 # Joint Prevention and Control Mechanism	51	[‘Joint Prevention and Control’, ‘Work Resumption’, ‘Hospital’, ‘Transportation’, ‘Nucleic Acid Testing’, ‘Asymptomatic Patient’, ‘Social Organization’]
3–2 # Informatization Helps Work Resumption	19	[‘Small Enterprises’, ‘Digitalization’, ‘Work Resumption’, ‘Supply Chain’, ‘Industry Chain’, ‘Informatization’, ‘Financial Institution’, ‘Internet’, ‘Volunteer Service’]
3–3 # Technology Assist Prevention	16	[‘Internet’, ‘Scientific and Technological Advance’, ‘Ministry of Science and Technology’, ‘Marketization’, ‘Technological Innovation’, ‘Ministry of Agriculture and Rural Affairs’, ‘Community Worker’, ‘Agricultural Production’]
3–4 # Market Regulation Protected by Law	16	[‘Service Industry’, ‘Agricultural Products’, ‘Ministry of Civil Affairs’, ‘Supply Chain’, ‘Poor Area’, ‘Economy and Society’, ‘Adjust Measures to Local Conditions’]
3–5 # Social Work for Vulnerable Population	12	[‘Nursing Facility’, ‘Civil Affairs’, ‘Pension Service’, ‘Poverty Alleviation’, ‘National Standard’, ‘Peasant Worker’, ‘Poor Area’, ‘Ministry of Construction’]
3–6 # Financial Investment	10	[‘Ministry of Commerce’, ‘Pedestrian Zone’, ‘Economy and Society’, ‘Human Resource’, ‘Management Responsibility’, ’Foreign Investment’, ‘Foreign-invested Enterprise’]
3–7 # Consumption Recovery	9	[‘Intellectual Property’, ‘High Quality’, ‘Service Industry’, ‘Public Service’, ‘Agricultural Products’, ’Economic and Social’, ‘Ministry of Tourism’, ‘Return to Work’]
3–8 # Psychological Counseling	7	[‘Social Work’, ‘Mental Health’, ‘Psychological Counseling’, ‘Immigration Officer’, ‘College Graduates’, ‘Transportation’, ‘Human Resources’, ‘Social Assistance’]
3–9 # Transportation	5	[‘Road Transport’, ‘Operator’, ‘Return to Work’, ‘Market Supervision Administration’, ‘Fair Competition’, ‘Consumer’, ‘Transportation’, ‘Daily Life’]

**Table 4:** Topic contents in Stage 4.

Cluster Topics	Number of Documents	High-Frequency Tagged Words
4–1 # Stabilising and Promoting Employment	85	[‘Human Resource’, ‘Ministry of Finance’, ‘Transportation’, ‘Production Safety’, ‘Department of Social Security’, ‘College Graduate’, ‘Informatization’, ’Ministry of Commerce’, ‘Vocational skill’]
4–2 # Medical Research	33	[‘Laboratory’, ‘Medical Institution’, ‘Nucleic acid testing reagent’, ’Biosafety’, ‘Health’, ‘Ministry of Education’, ‘Institution of Higher Education’, ‘Joint Prevention and Control’]
4–3 # Medical and Health Service	27	[‘Public Health’, ‘Medical Service’, ‘Infection’, ‘Disinfectant’, ‘Internet’, ‘Ministry of Finance’, ‘Health Care’, ‘Disease Prevention’, ‘Medical Security’, ‘Inspection’]
4–4 # Environment and Market Regulation	24	[‘Yangtze River Basin’, ‘Natural Resource’, ‘Regulator’, ‘Network Security’, ‘Internet’, ‘Agriculture and rural area’, ‘Crack Down’, ‘Market Regulation’, ‘Eco-environment’]
4–5 # Regular Prevention and Control	22	[‘Joint Prevention and Control’, ‘Wear Mask’, ‘Prevention and Control Measure’, ‘Body Temperature Monitoring’, ‘Individual Protection’, ‘Environmental Sanitation’, ‘Health Monitoring’]
4–6 # Small Enterprise Financial Service	10	[‘Small Enterprise’, ‘Financial Institution’, ‘Commercial Bank’, ‘Pilot Zone’, ‘Financial Service’, ‘Financial Guarantee’, ‘Individual Businesses’, ‘Credit’]
4–7 # Education for Poverty Alleviation	9	[‘Compulsory Education’, ‘Poverty Alleviation’, ‘Poor Family’, ‘Ministry of Education’, ‘Middle and Primary Schools’, ‘Civil Affairs’, ‘Human Resource’, ‘The Poor’, ’Social Security’]

## Data Availability

The data presented in this study are available on request from the corresponding author. The data are not publicly available due to privacy restrictions.

## References

[1] Christensen T., Laegreid P. (2020). Balancing Governance Capacity and Legitimacy: How the Norwegian Government Handled the COVID-19 Crisis as a High Performer. Public Adm. Rev..

[2] Woo J.J. (2020). Policy Capacity and Singapore’s Response to the COVID-19 Pandemic. Policy Soc..

[3] Capano G. (2020). Policy Design and State Capacity in the COVID-19 Emergency in Italy: If You Are Not Prepared for the (Un) Expected, You Can Be Only What You Already Are. Policy Soc..

[4] Tulenko K., Vervoort D. (2020). Cracks in the System: The Effects of the Coronavirus Pandemic on Public Health Systems. Am. Rev. Public Adm..

[5] Kirlin J. (2020). COVID-19 Upends Pandemic Plan. Am. Rev. Public Adm..

[6] Cairney P. (2021). The UK Government’s COVID-19 Policy: Assessing Evidence-Informed Policy Analysis in Real Time. Br. Polit..

[7] Sridhar D. This is what you Should be Demanding from Your Government to Contain the Virus. http://www.theguardian.com/commentisfree/2020/may/04/eight-lessons-controlling-coronavirus-east-asian-nations-pandemic-public-health.

[8] Cheng Y., Yu J., Shen Y., Huang B. (2020). Coproducing Responses to COVID-19 with Community-Based Organizations: Lessons from Zhejiang Province, China. Public Adm. Rev..

[9] He A.J., Shi Y., Liu H. (2020). Crisis Governance, Chinese Style: Distinctive Features of China’s Response to the Covid-19 Pandemic. Policy Des. Pract..

[10] Ahmad E. (2020). Multilevel Responses to Risks, Shocks and Pandemics: Lessons from the Evolving Chinese Governance Model. J. Chin. Gov..

[11] Liu Y., Saltman R.B. (2020). Policy Lessons From Early Reactions to the COVID-19 Virus in China. Am. J. Public Health.

[12] Peng F., Tu L., Yang Y., Hu P., Wang R., Hu Q., Cao F., Jiang T., Sun J., Xu G. (2020). Management and Treatment of COVID-19: The Chinese Experience. Can. J. Cardiol..

[13] Zhang X., Luo W., Zhu J. (2021). Top-Down and Bottom-Up Lockdown: Evidence from COVID-19 Prevention and Control in China. J. Chin. Polit. Sci..

[14] Mei C. (2020). Policy Style, Consistency and the Effectiveness of the Policy Mix in China’s Fight against COVID-19. Policy Soc..

[15] Liu P., Zhong X., Yu S. (2020). Striking a Balance between Science and Politics: Understanding the Risk-Based Policy-Making Process during the Outbreak of COVID-19 Epidemic in China. J. Chin. Gov..

[16] Yang K. (2020). Unprecedented Challenges, Familiar Paradoxes: COVID-19 and Governance in a New Normal State of Risks. Public Adm. Rev..

[17] Hu Q., Zhang H., Kapucu N., Chen W. (2020). Hybrid Coordination for Coping with the Medical Surge from TheCOVID-19 Pandemic:Paired AssistancePrograms in China. Public Adm. Rev..

[18] Li Y., Chandra Y., Kapucu N. (2020). Crisis Coordination and the Role of Social Media in Response to COVID-19 in Wuhan, China. Am. Rev. Public Adm..

[19] Day M. (2020). Covid-19: Four Fifths of Cases Are Asymptomatic, China Figures Indicate. BMJ.

[20] Krstic K., Westerman R., Chattu V.K., Ekkert V.N., Jakovljevic M. (2020). Corona-Triggered Global Macroeconomic Crisis of the Early 2020s. Int. J. Environ. Res. Public. Health.

[21] Roosa K., Lee Y., Luo R., Kirpich A., Rothenberg R., Hyman J.M., Yan P., Chowell G. (2020). Real-Time Forecasts of the COVID-19 Epidemic in China from February 5th to February 24th, 2020. Infect. Dis. Model..

[22] Sun T.-T., Tao R., Su C.-W., Umar M. (2021). How Do Economic Fluctuations Affect the Mortality of Infectious Diseases?. Front. Public Health.

[23] Jakovljevic M., Sugahara T., Timofeyev Y., Rancic N. (2020). Predictors of (in) Efficiencies of Healthcare Expenditure Among the Leading Asian Economies—Comparison of OECD and Non-OECD Nations. Risk Manag. Healthc. Policy.

[24] Xie J., Tong Z., Guan X., Du B., Qiu H., Slutsky A.S. (2020). Critical Care Crisis and Some Recommendations during the COVID-19 Epidemic in China. Intensive Care Med..

[25] Davidovitz M., Cohen N., Gofen A. (2021). Governmental Response to Crises and Its Implications for Street-Level Implementation: Policy Ambiguity, Risk, and Discretion during the COVID-19 Pandemic. J. Comp. Policy Anal..

[26] Shi Y., Jang H.S., Keyes L., Dicke L. (2020). Nonprofit Service Continuity and Responses in the Pandemic: Disruptions, Ambiguity, Innovation, and Challenges. Public Adm. Rev..

[27] Dong Q., Lu J. (2020). In the Shadow of the Government: The Chinese Nonprofit Sector in the COVID-19 Crisis. Am. Rev. Public Adm..

[28] Cepiku D., Giordano F., Bovaird T., Loeffler E. (2021). New Development: Managing the Covid-19 Pandemic-from a Hospital-Centred Model of Care to a Community Co-Production Approach. Public Money Manag..

[29] Sancino A., Garavaglia C., Sicilia M., Braga A. (2020). New Development: Covid-19 and Its Publics-Implications for Strategic Management and Democracy. Public Money Manag..

[30] Zhao T., Wu Z. (2020). Citizen-State Collaboration in Combating COVID-19 in China: Experiences and Lessons From the Perspective of Co-Production. Am. Rev. Public Adm..

[31] Zhang S., Wang Z., Chang R., Wang H., Xu C., Yu X., Tsamlag L., Dong Y., Wang H., Cai Y. (2020). COVID-19 Containment: China Provides Important Lessons for Global Response. Front. Med..

[32] Wardman J.K. (2020). Recalibrating Pandemic Risk Leadership: Thirteen Crisis Ready Strategies for COVID-19. J. Risk Res..

[33] An B.Y., Tang S.-Y. (2020). Lessons From COVID-19 Responses in East Asia: Institutional Infrastructure and Enduring Policy Instruments. Am. Rev. Public Adm..

[34] Hick R., Murphy M.P. (2021). Common Shock, Different Paths? Comparing Social Policy Responses to COVID-19 in the UK and Ireland. Soc. Policy Adm..

[35] Beland D., Marchildon G.P., Medrano A., Rocco P. (2021). COVID-19, Federalism, and Health Care Financing in Canada, the United States, and Mexico. J. Comp. Policy Anal..

[36] Weng S.-H., Ni A.Y., Ho A.T.-K., Zhong R.-X. (2020). Responding to the Coronavirus Pandemic: A Tale of Two Cities. Am. Rev. Public Adm..

[37] Downey D.C., Myers W.M. (2020). Federalism, Intergovernmental Relationships, and Emergency Response: A Comparison of Australia and the United States. Am. Rev. Public Adm..

[38] Yan B., Zhang X., Wu L., Zhu H., Chen B. (2020). Why Do Countries Respond Differently to COVID-19? A Comparative Study of Sweden, China, France, and Japan. Am. Rev. Public Adm..

[39] Fowler L., Kettler J.J., Witt S.L. (2021). Pandemics and Partisanship: Following Old Paths into Uncharted Territory. Am. Polit. Res..

[40] Baccini L., Brodeur A. (2021). Explaining Governors’ Response to the COVID-19 Pandemic in the United States. Am. Polit. Res..

[41] Brousselle A., Brunet-Jailly E., Kennedy C., Phillips S.D., Quigley K., Roberts A. (2020). Beyond COVID-19: Five Commentaries on Reimagining Governance for Future Crises and Resilience. Can. Public Adm. Adm. Publique Can..

[42] Di Mascio F., Natalini A., Cacciatore F. (2020). Public Administration and Creeping Crises: Insights From COVID-19 Pandemic in Italy. Am. Rev. Public Adm..

[43] Simon H.A. (1996). Designing Organizations for an Information-Rich World. Int. Libr. Crit. Writ. Econ..

[44] Turrini A., Cristofoli D., Valotti G. (2020). Sense or Sensibility? Different Approaches to Cope with the COVID-19 Pandemic. Am. Rev. Public Adm..

[45] Janssen M., van der Voort H. (2020). Agile and Adaptive Governance in Crisis Response: Lessons from the COVID-19 Pandemic. Int. J. Inf. Manag..

[46] Jones B.D. (1994). Reconceiving Decision-Making in Democratic Politics: Attention, Choice, and Public Policy.

[47] Saurugger S., Terpan F. (2016). Do Crises Lead to Policy Change? The Multiple Streams Framework and the European Union’s Economic Governance Instruments. Policy Sci..

[48] Huang C., Yang C., Su J. (2018). Policy Change Analysis Based on “Policy Target–Policy Instrument” Patterns: A Case Study of China’s Nuclear Energy Policy. Scientometrics.

[49] Blei D.M., Ng A.Y., Jordan M.I. (2003). Latent Dirichlet Allocation. J. Mach. Learn. Res..

[50] Blei D.M. (2012). Probabilistic Topic Models. Commun. ACM.

[51] Griffiths T.L., Steyvers M. (2004). Finding Scientific Topics. Proc. Natl. Acad. Sci. USA.

[52] Ansell C., Sorensen E., Torfing J. (2020). The COVID-19 Pandemic as a Game Changer for Public Administration and Leadership? The Need for Robust Governance Responses to Turbulent Problems. Public Manag. Rev..

[53] Kettl D.F. (2020). States Divided: The Implications of American Federalism for COVID-19. Public Adm. Rev..

[54] Gao X., Yu J. (2020). Public Governance Mechanism in the Prevention and Control of the COVID-19: Information, Decision-Making and Execution. J. Chin. Gov..

[55] Khan M., Roy P., Matin I., Rabbani M., Chowdhury R. (2021). An Adaptive Governance and Health System Response for the COVID-19 Emergency. World Dev..

[56] Andrew S.A., Kendra J.M. (2012). An Adaptive Governance Approach to Disaster-Related Behavioural Health Services. Disasters.

[57] Wolff S., Ladi S. (2020). European Union Responses to the Covid-19 Pandemic: Adaptability in Times of Permanent Emergency. J. Eur. Integr..

[58] Hizbaron D.R., Ruslanjari D., Mardiatno D. (2021). Amidst Covid-19 Pandemic: An Adaptive Disaster Governance in Yogyakarta, Indonesia. Soc. Sci..

[59] Scholz J., Stiftel B. (2005). Adaptive Governance and Water Conflicts: New Institutions for Collaborative Planning.

[60] Zhang H., Zhang X., Comfort L., Chen M. (2016). The Emergence of an Adaptive Response Network: The April 20, 2013 Lushan, China Earthquake. Saf. Sci..

[61] Schmidt V.A. (2020). Theorizing Institutional Change and Governance in European Responses to the Covid-19 Pandemic. J. Eur. Integr..

[62] Moloney K., Moloney S. (2020). Australian Quarantine Policy: From Centralization to Coordination with Mid-Pandemic COVID-19 Shifts. Public Adm. Rev..

[63] Nohrstedt D. (2005). External Shocks and Policy Change: Three Mile Island and Swedish Nuclear Energy Policy. J. Eur. Public Policy.

[64] Nohrstedt D., Weible C.M. (2010). The Logic of Policy Change after Crisis: Proximity and Subsystem Interaction. Risk Hazards Crisis Public Policy.

[65] Walgrave S., Varone F. (2008). Punctuated Equilibrium and Agenda-Setting: Bringing Parties Back in: Policy Change after the Dutroux Crisis in Belgium. Governance.

